# Antimicrobial Activity of Cellulose Based Materials

**DOI:** 10.3390/polym14040735

**Published:** 2022-02-14

**Authors:** Nicoleta Sorina Nemeş, Cristina Ardean, Corneliu Mircea Davidescu, Adina Negrea, Mihaela Ciopec, Narcis Duţeanu, Petru Negrea, Cristina Paul, Daniel Duda-Seiman, Delia Muntean

**Affiliations:** 1 Renewable Energy Research Institute-ICER, Politehnica University of Timisoara, 138 Gavril Musicescu Street, 300501 Timisoara, Romania; nicoleta.nemes@upt.ro; 2 Faculty of Industrial Chemistry and Environmental Engineering, Politehnica University of Timişoara, 2 Piaţa Victoriei, 300006 Timisoara, Romania; cristina.ardean@student.upt.ro (C.A.); adina.negrea@upt.ro (A.N.); mihaela.ciopec@upt.ro (M.C.); petru.negrea@upt.ro (P.N.); cristina.paul@upt.ro (C.P.); 3 Department of Cardiology, “Victor Babeş” University of Medicine and Pharmacy Timişoara, 2 Piata Eftimie Murgu, 300041 Timisoara, Romania; dannymduda@gmail.com; 4 Multidisciplinary Research Center on Antimicrobial Resistance, Department of Microbiology, “Victor Babeş” University of Medicine and Pharmacy, 2 Eftimie Murgu Square, 300041 Timisoara, Romania; muntean.delia@umft.ro

**Keywords:** cellulose, antimicrobial activities, functionalized materials, cellulose derivatives

## Abstract

Biomaterials available for a wide range of applications are generally polysaccharides. They may have inherent antimicrobial activity in the case of chitosan. However, in order to have specific functionalities, bioactive compounds must be immobilized or incorporated into the polymer matrix, as in the case of cellulose. We studied materials obtained by functionalizing cellulose with quaternary ammonium salts: dodecyl-trimethyl-ammonium bromide (DDTMABr), tetradecyl-trimethyl-ammonium bromide (TDTMABr), hexadecyl-trimethyl ammonium chloride (HDTMACl), some phosphonium salts: dodecyl-triphenyl phosphonium bromide (DDTPPBr) and tri n-butyl-hexadecyl phosphonium bromide (HDTBPBr) and extractants containing sulphur: 2-mercaptobenzothiazole (MBT) and thiourea (THIO). Cel-TDTMABr material, whose alkyl substituent chain conformation was shortest, showed the best antimicrobial activity for which, even at the lowest functionalization ratio, 1:0.012 (w:w), the microbial inhibition rate is 100% for *Staphylococcus aureus, Escherichia coli,* and *Candida albicans*. Among the materials obtained by phosphonium salt functionalization, Cel-DDTPPBr showed a significant bactericidal effect compared to Cel-HDTBPBr. For instance, to the same functionalization ratio = 1:0.1, the inhibition microbial growth rate is maximum in the case of Cel-DDTPPBr for *Staphylococcus aureus, Escherichia coli,* and *Candida albicans.* At the same time, for the Cel-HDTBPBr material, the total bactericidal effect is not reached even at the functionalization ratio 1:0.5. This behavior is based on the hydrophobicity difference between the two extractants, DDTPPBr and HDTBPBr. Cel-MBT material has a maximum antimicrobial effect upon *Staphylococcus aureus, Escherichia coli,* and *Candida albicans* at functionalized ratio = 1:0.5. Cel-THIO material showed a bacteriostatic and fungistatic effect, the inhibition of microbial growth being a maximum of 76% for *Staphylococcus aureus* at the functionalized ratio = 1:0.5. From this perspective, biomaterials obtained by SIR impregnation of cellulose can be considered a benefit to be used to obtain biomass-derived materials having superior antimicrobial properties versus the non-functional support.

## 1. Introduction

Cellulose is one of the major components of plant cell walls. Cellulose is mainly obtained from natural resources such as plant materials: grass (bamboo), straw (wheat, rice, barley), leaves (pineapple, palm), fibers (flax, jute, hemp, sugar cane, sisal) [1,2], wood and wood residues such as wood chips or branches [1,3,4,5,6], marine animals [6,7], fungi, algae and bacteria [6,8,9,10,11,12].

Regardless of the source [13,14], cellulose can be characterized as a linear homopolysaccharide with high molecular weight, consisting of monomer units arranged alternately with an angle of 180℃ between them, linked by β-1,4 bonds [15,16]. The repeating segment is a glucose dimer known as cellobiose [6,14,17]. Each monomer unit, identified as an anhydrous-glucose unit, has three hydroxyl groups, giving cellulose the most important properties and determining its microfibrillated structure [2,9,14] and the hierarchical organization in crystalline and amorphous regions and the extremely cohesive structure of the molecule [7,18].

Each cellulose chain has a directional asymmetry of its molecular axis with respect to the ends. One end has reduced chemical functionality due to the hemiacetal unit being a COC-shaped etheric bridge. The other end has “pendant” type hydroxyl groups with no reduction properties [14]. However, hydroxyl groups play a major role in forming covalent bonds with various reactants [19], which increase the chemical activity of cellulose and thus the antibacterial effect.

Due to the biocompatibility and biodegradability of cellulose [20], but also through the production of a wide range of cellulose derivatives [21], this biopolymer is used in various technological fields: in the food industry, it is used as a gelling or stabilizing agent [20,22], while in cosmetics it is used as a scrub or water retention agent [16,23,24], emulsifier [22,25], stabilizer of suspensions in paste and cream [24]. Other applications are in the paper industry [23], textile industry [16,23], and leather [26] or as combustible [27]. Various materials made from bacterial cellulose and polymeric compounds were tested to obtain artificial bones, cartilage, or other medical devices [23,28]. Of all the known types, the most studied form of cellulose for obtaining materials with antibacterial properties was cellulose with Ag nanoparticles, considering the antibacterial potential of Ag nanoparticles [11,29,30,31].

Microcrystalline cellulose (like the cellulose used in this study, Avicel PH 101) is a partially depolymerized pure cellulose synthesized from the α-cellulose precursor (type Iβ). This type of cellulose is considered a remarkable excipient. It remains the most widely used and the strongest dry binder, disintegrating tablet coating, absorbent, lubricant, and non-stick material [32]. Cellulose has good water purifying effects [16,33] because it has many free -OH groups on the chain, allowing the efficient removal of metal ions and organic matter from water [33] having an excellent chelating effect. However, due to its low water solubility and relatively low chemical reactivity, the use of unmodified cellulose as a flocculant is limited [34]. Modified, water-soluble (hydrophilic) cellulose plays a very important role as a potential substitute for oil-based flocculants [35]. Some cellulose derivatives have already been successfully tested to remove suspended solids. There is also a growing interest in developing low-cost biomass (cellulosic) absorbents for the treatment of colour-contaminated wastewater (agricultural, industrial, municipal waste). Thus, anionic sodium carboxymethylcellulose, prepared from palm agricultural waste, has been tested as an environmentally friendly flocculant coupled with aluminum sulfate as a coagulant to remove turbidity in drinking water treatment [34,36].

As was said, natively, cellulose has a relatively low reactivity [5]. Therefore, introducing new functional groups on the cellulose structure can increase the polarity and hydrophilicity of its surface [23], which can increase the adsorption of polar compounds and the selectivity of cellulose for the targeted pollutant. However, the chemical modification of this natural polymer is slightly difficult due to its relatively low reactivity. This is also influenced by the many hydrogen bonds that decrease the potential solubility in most common solvents [23]. Typically, the modification of cellulose fibers is achieved by heterogeneous synthesis [23,37], which can lead to some unexpected by-products [37] or the decomposition of cellulose. Although obtaining materials with antibacterial properties based on cellulose by simply mixing with antibacterial agents could be a way to induce antibacterial activity to the biocellulose, antibacterial agents being leached during use and thus bioactivity will decrease. That is why it is preferable to obtain cellulose derivatives by grafting chains with antibacterial activity on the cellulose skeleton, as we will show in this paper. Quaternary ammonium salts or polymers containing quaternary ammonium groups are well known for their increased antibacterial activity [5,21,38]. On the other hand, by impregnating cellulose with various extractants containing active groups (SIR Method) [39,40,41,42], the use of harsh reaction conditions is avoided, which is in accordance with the principles of green chemistry.

## 2. Materials and Methods

### 2.1. Obtaining and Characterizing Materials

The cellulose used to obtain the materials used in the present study (powder, Avicel PH–101, Sigma-Aldrich, Merck, KGaA, Darmstadt, Germany) was used as a support, and dodecyl-trimethyl-ammonium bromide (DDTMABr—Acros Organics, Waltham, MA, USA), tetradecyl-trimethyl-ammonium bromide (TDTMABr—ThermoFisher, Waltham, MA, USA, purity 98%), hexadecyl-trimethyl ammonium chloride (HDTMACl—ThermoFisher, Germany, purity 98%), dodecyl-triphenyl phosphonium bromide (DDTPPBr—ThermoFisher, Germany purity 98%), tri n-butyl-hexadecyl phosphonium bromide (HDTBPBr—ThermoFisher, Germany, purity 98%), 2-mercaptobenzothiazole (MBT—Janssen Chemistry, Bucharest, Romania), thiourea (THIO—Fluka AG, Charlotte, NC, USA). The method used to obtain the materials was the dry method SIR (Solvent Impregnated Resin), by which the support is functionalized by impregnation [41]. The support and the extractant (dissolved in distilled water) are in contact for 24 h at 323 K. Then, the obtained sample is filtered, and the material obtained is dried in an oven (POLENKO SLW53, Poland). Materials were obtained in which the support:extractant (w/w) ratio of is different, namely: 1:0.012; 1:0.025; 1:0.050; 1:0.075; 1:0.1; 1:0.2; 1:0.3; 1:0.4; 1:0.5. The obtained material was characterized by scanning electron microscopy, SEM, and X-ray energy dispersion (EDX), using the X-ray energy dispersion spectrometer and the FEI Quanta FEG 250 instrument (FEI, Hillsboro, OR, USA).

### 2.2. Bacterial Culture Preparation

(a) Microbial cultures preparation, in order to establish the optimal cellulose: extractant ratio

Bacterial cultures were obtained by inoculating a non-selective solid nutrient medium into which the test material was incorporated, then poured into Petri dishes, with a bacterial suspension of approx. 1 × 10^8^ UFC/mL (0,5 Mc Farland). Completely dehydrated culture medium was used for all seedings, Plate Count Agar-produced by Merck (Peptone from Casein 5.0 g/L; Yeast Extract 2.5 g/L; D (+)-Glucose 1.0 g/L; Agar 14.0 g/L).

In each set of experiments, a Petri dish containing the control sample (M0-control sample) was seeded, consisting of culture medium and 1 mL of bacterial inoculum, and a Petri dish containing culture medium, bacterial inoculum, and non-functionalized support material, in solid-state (M1-cellulose (Cel)). 

Each functionalized material (containing both supporting and extracting material) to be tested was analysed in all functionalization ratios: 1:0.012; 1:0.025; 1:0.050; 1:0.075; 1:0.1; 1:0.2; 1:0.3; 1:0.4; 1:0.5. An amount of non-functionalized or functionalized solid material of about 0.2 g was used in each Petri dish, which was distributed as evenly as possible in the culture medium and 1 mL of bacterial inoculum (approx. 1 × 10^8^ UFC/mL), in an amount of culture medium of about 15 mL.

All inoculations were performed with 3 repetitions. The plates were incubated for 48 h at 310 K. Subsequently, the colonies developed on the surface of the Petri dishes were counted using an automatic colony counter produced by YUL (Flash&Go, YUL Instruments SA, Barcelona, Spain).

(b) Microbial cultures preparation for further tests of antimicrobial effect of the synthesized materials on the reference strains

In order to test the antimicrobial effect of the synthesized materials on the reference microbial strains, both the optimal support:extractant ratios determined in the first stage and other support:extractant ratios were considered so that the efficiency of the functionalization ratio can be monitored. In order to establish the efficacy of the functionalized material, microbiological control tests were performed on 1 × 10^5^ CFU/mL of reference microbial strains (Thermo Scientific, Waltham, MA, USA). To highlight these effects on Gram-negative bacteria, strains of *Escherichia coli ATCC 25922* and *Pseudomonas aeruginosa ATCC 27853* were studied, while the effect of the materials on Gram-positive bacteria was studied on the strain of *Staphylococcus aureus ATCC 25923*. *Candida albicans* strain *ATCC 10231* was used to observe the antifungal activity. All determinations were performed with 3 repetitions. The plates were incubated for 48 h at 310 K. The number of colonies developed on the surface of the work plates was determined using the automatic colony counter from YUL instruments (YUL Instruments SA, Barcelona, Spain). The efficiency of the functionalized material on the reference strains was expressed as the rate of inhibition of microbial growth [43,44,45], calculated as the ratio between the number of colony-forming units on functionalized and non-functionalized material, expressed as a percentage ratio, according to the equation:

(1) $$inhibition\;rate=\frac{\left[UFC_{control}-UFC_{test}\right]}{UFC_{control}}\times 100$$

*UFC_control_* = number of colonies on the control plate
*UFC_test_* = the number of colonies on the test plate

Using this method, the optimal support:extractant ratio was established, representing the optimum ratio at which no microbial growth was observed or the case when the rate of inhibition of microbial growth was the highest. 

Total bactericidal effect was considered where the rate of inhibition of bacterial growth was 100%.

## 3. Results and Discussion

### 3.1. Characterization of Materials Obtained by Cellulose Functionalizing 

Obtained materials were characterized by scanning electron microscopy, SEM, X-ray energy dispersion, EDX and infrared spectroscopy with Fourier transform, FT-IR to highlight the presence of pending groups N, P, and S of the extractant (DDTMABr, TDTMABr, HDTMACl, DDTPPBr, HDTBPBr, MBT and THIO) on the support surface (Cel).

- Scanning electron microscopy, SEM

**Figure 1:**
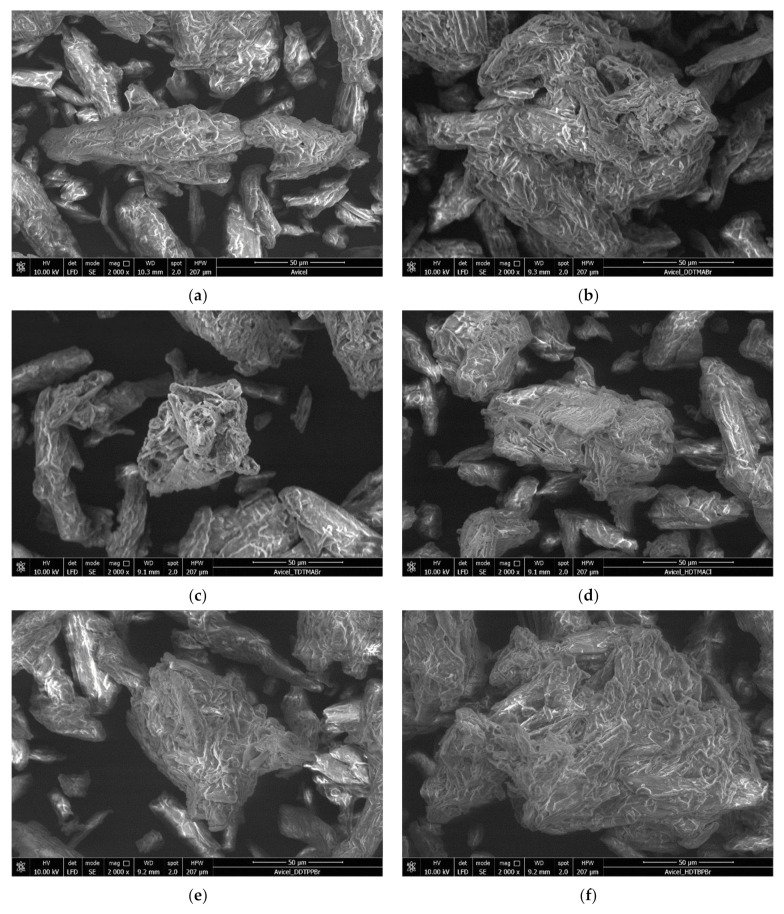

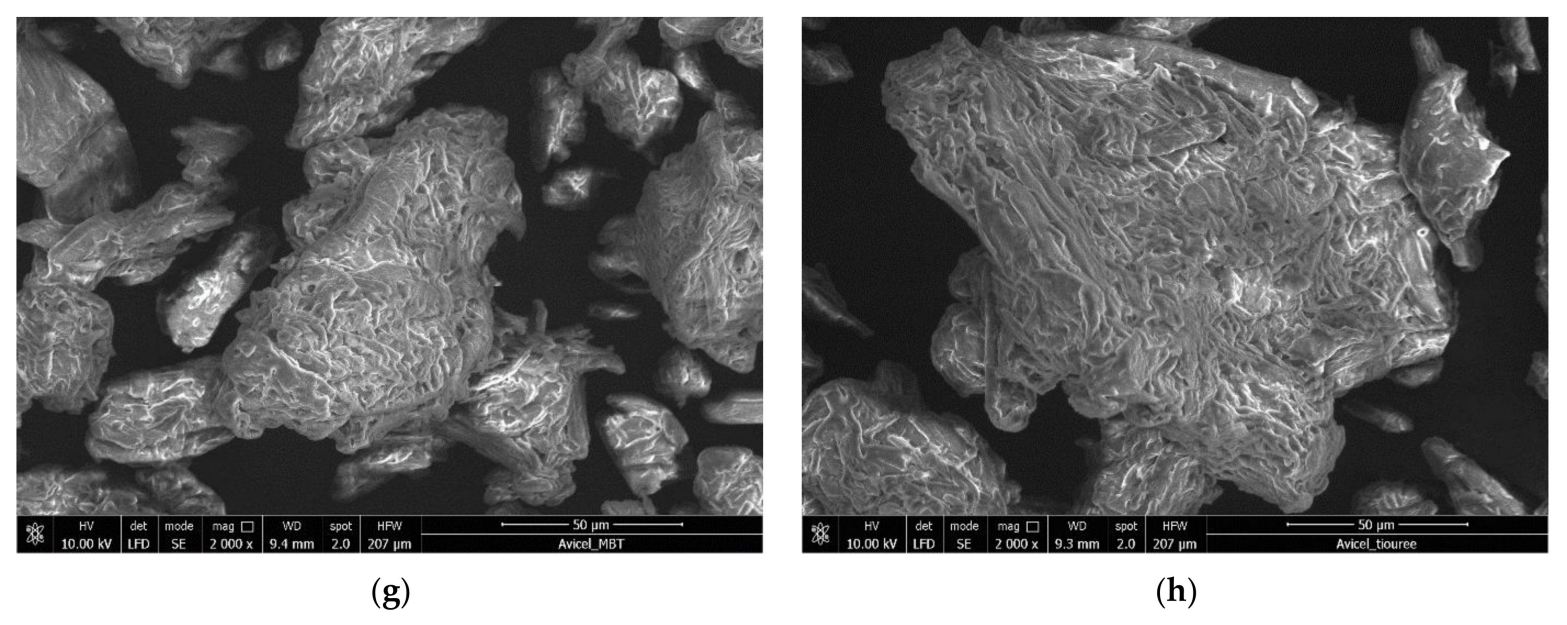
Scanning electron microscopy, SEM. (**a**). Cellulose (Cel); (**b**). Cel-DDTMABr; (**c**). Cel-TDTMABr; (**d**). Cel-HDTMACl; (**e**). Cel-DDTPPBr; (**f**). Cel-HDTBPBr; (**g**). Cel-MBT; (**h**). Cel-THIO.

To highlight the morphology of the support surface (cellulose), as well as of the materials obtained by impregnation by functionalization with the extractants DDTMABr, TDTMABr, HDTMACl, DDTPPBr, HDTBPBr, MBT, and THIO, the probes were analyzed by scanning electron microscopy operated at 10 kV and a magnification of 2000× (obtained micrograph being presented in Figure 1). The assays were performed in a low vacuum to prevent the sample’s destruction. 

It can be seen from Figure 1 that for all obtained materials, cellulose fibers are more agglomerated due to the functionalization. It was observed that the obtained materials have more slender fibers. However, the major structural difference between control and extractants-treated cellulose fibers was not clearly observed [46].

This morphological change is attributed to the swelling and partial disintegration of the cellulosic fibers during the surface modification process, followed by the insertion of amine moieties that cover the surface of the fibers [47,48]. Nevertheless, analogous micro-sized fibers with a rough and rod-shaped surface microstructure are revealed for samples based on microcrystalline cellulose, demonstrating the homogenous surface functionalization without much variation of surface morphology.

b. X-ray energy dispersion, EDX

**Figure 2:**
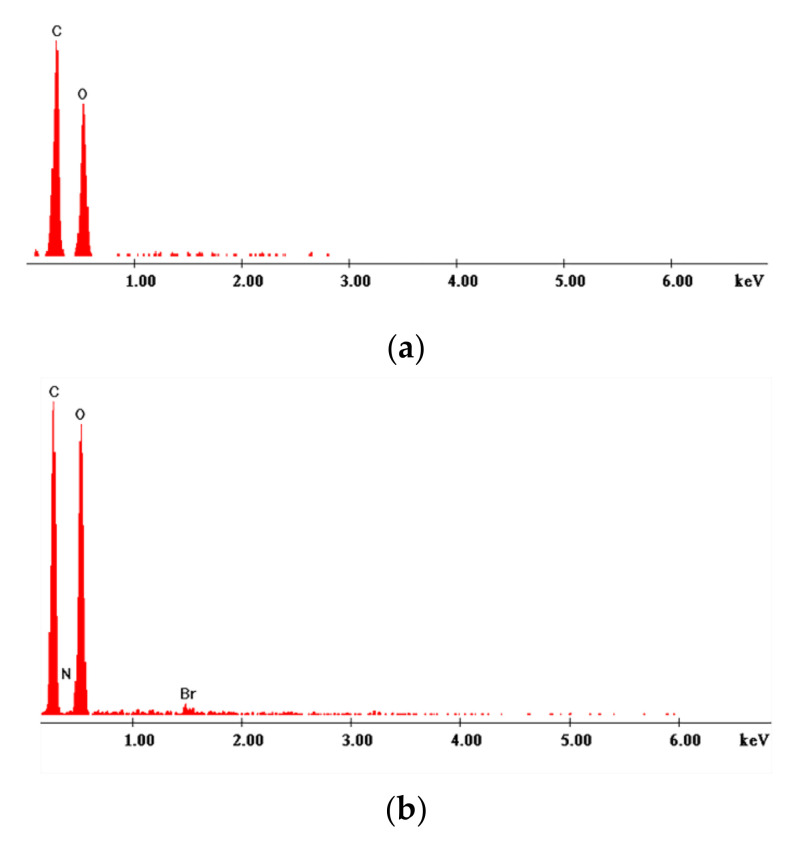

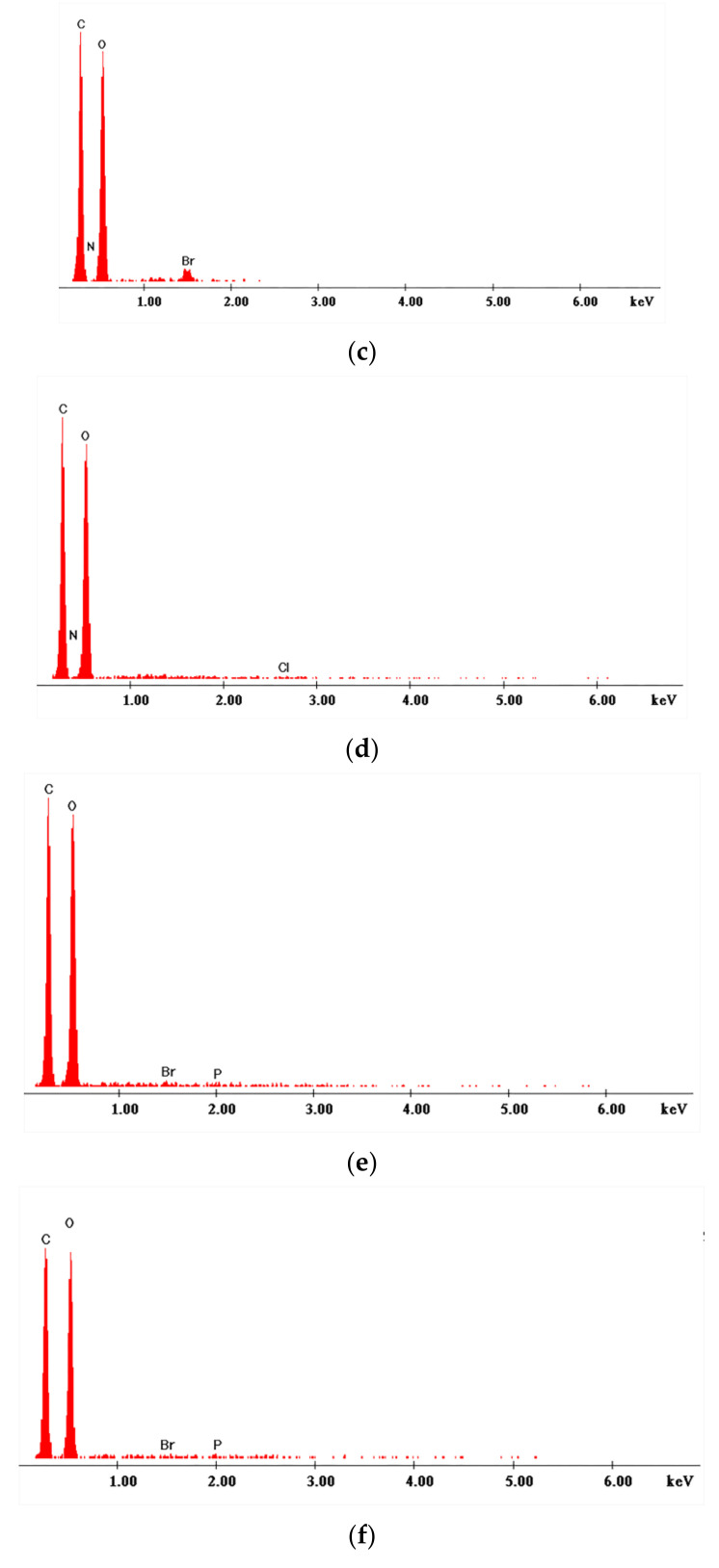

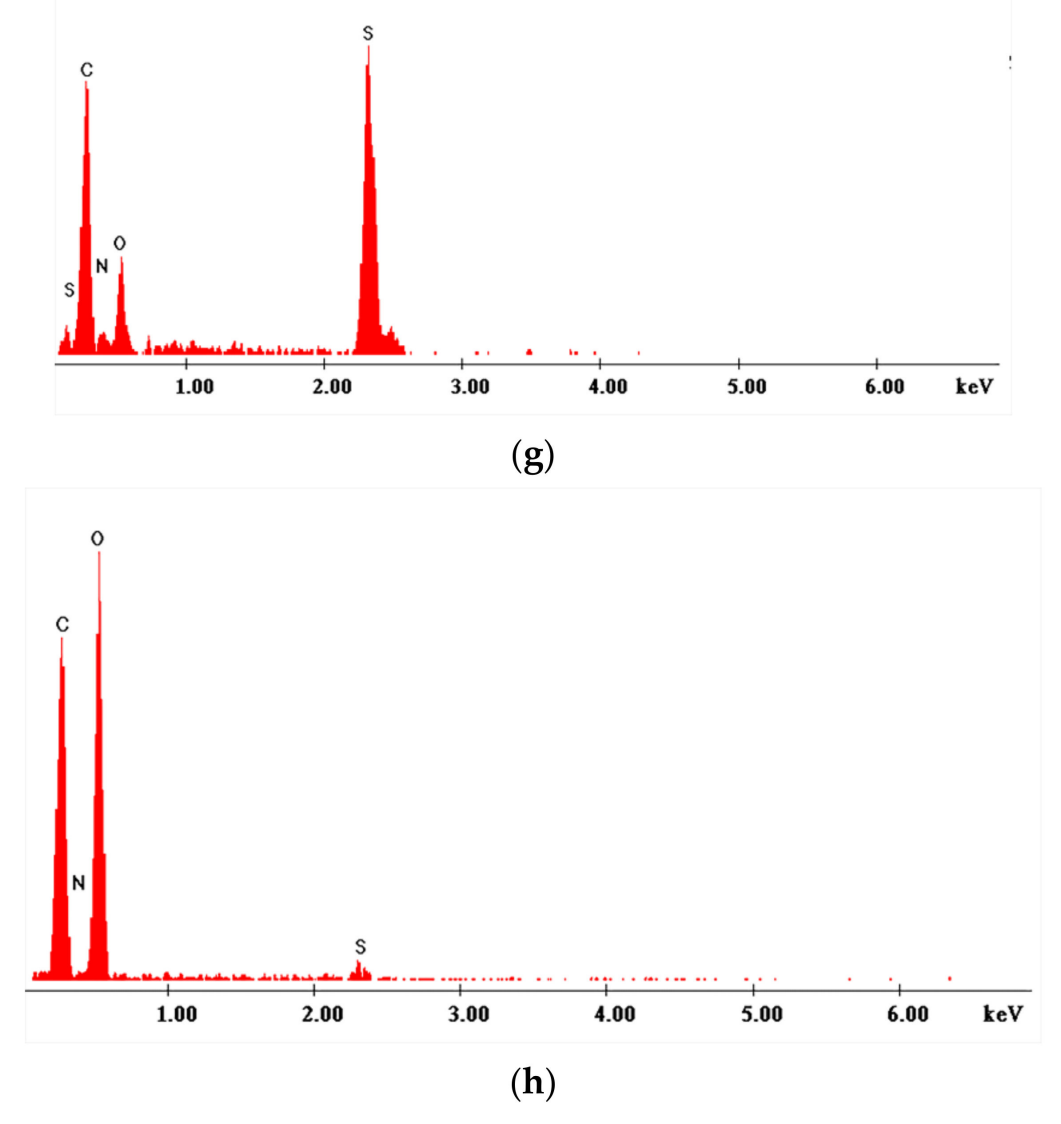
X-ray energy dispersion spectra recorded for pure cellulose and for prepared materials. (**a**). Cellulose (Cel); (**b**). Cel-DDTMABr; (**c**). Cel-TDTMABr; (**d**). Cel-HDTMACl; (**e**). Cel-DDTPPBr; (**f**). Cel-HDTBPBr; (**g**). Cel-MBT; (**h**). Cel-THIO.

The EDX spectra for support, Cel, and the obtained materials are presented in Figure 2.

Figure 2a shows the peaks specific to the composition of cellulose, namely C and O. Figure 2b–h shows the specific EDX spectra representing the specific peaks of the elements present in the composition of cellulose and the extractants. Thus, in Figure 2b,c there are drawn the specific peaks of the DDTMABr and TDTMABr extractants, associated with the presence of Br and N atoms, and in Figure 2d, in addition to the specific peak of N, there is also the specific peak in case of Cl. Figure 2e,f show specific P and Br peaks, elements that are present in the DDTPPBr and HDTBPBr extractants. In Figure 2g,h. specific S and N peaks appear, elements that are present in the MBT and THIO extractants. These observations reveal a successful functionalization of the natural polymer with thiourea extractant [49].

c. Infrared spectroscopy with Fourier transform, FT-IR

**Figure 3:**
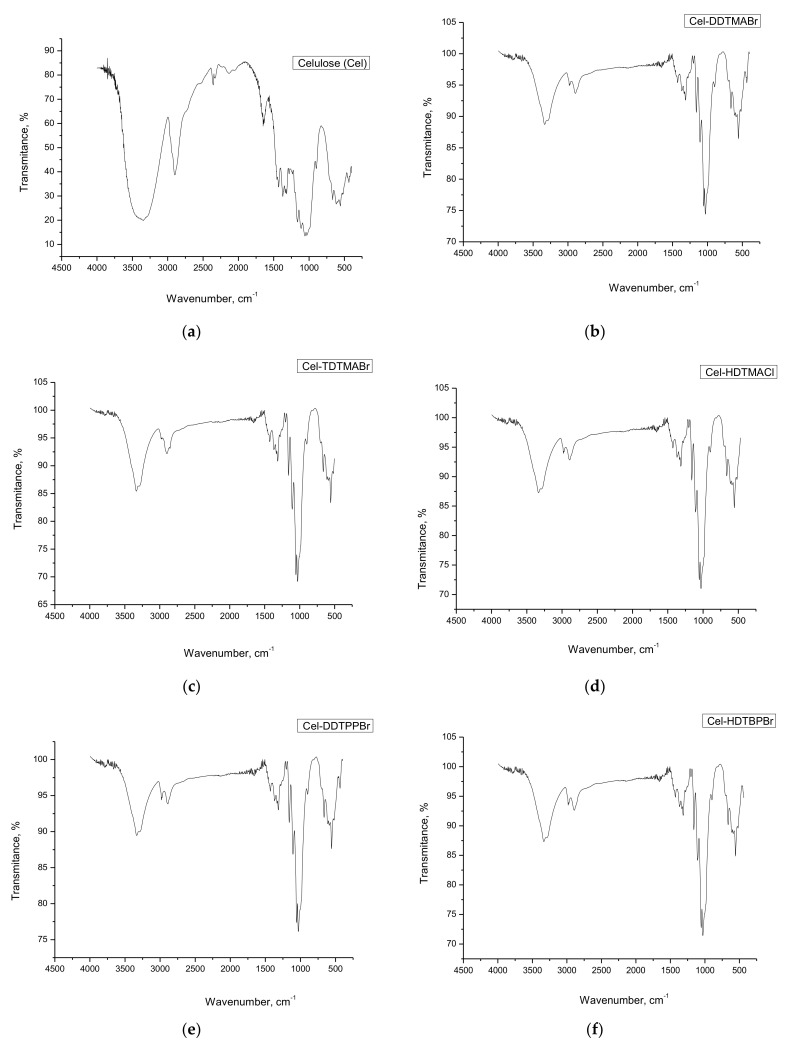

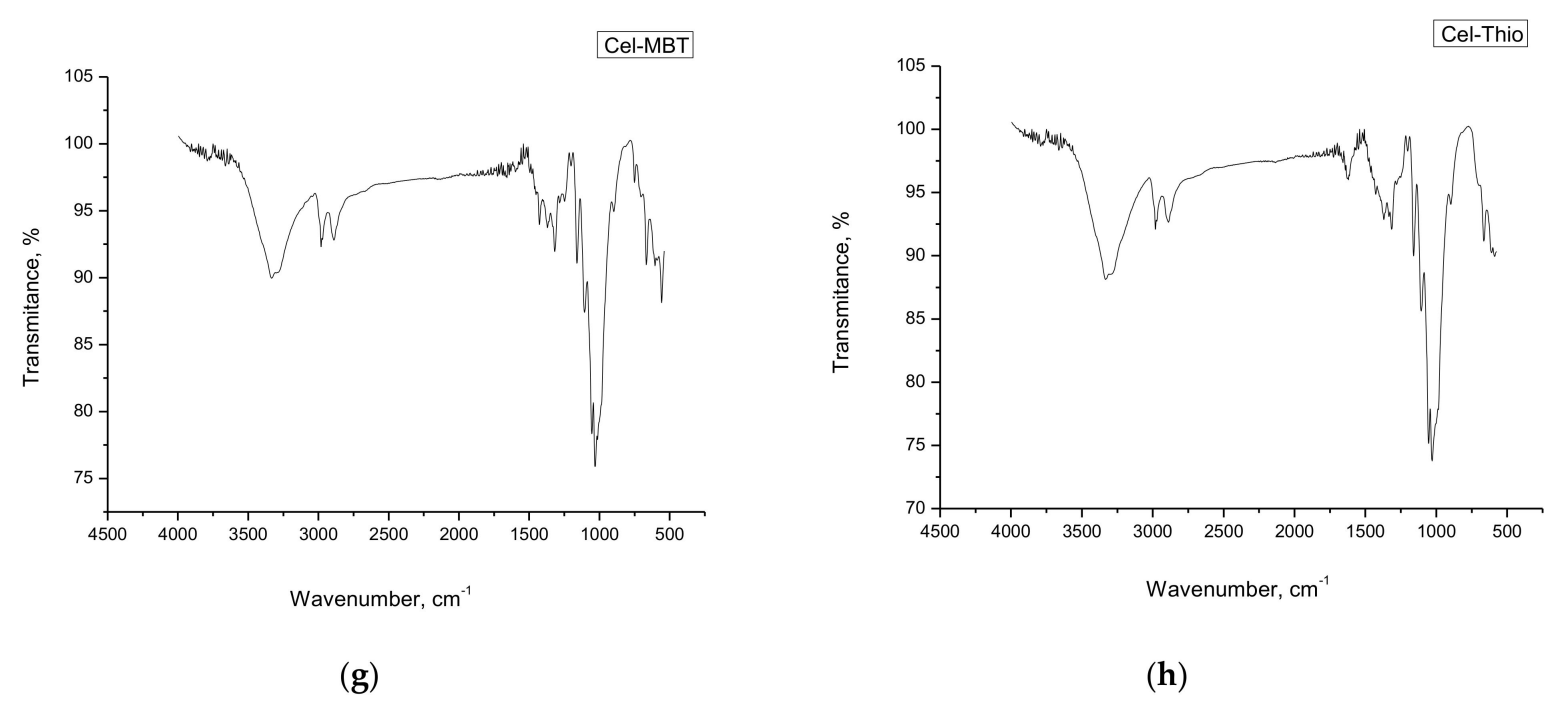
FT-IR spectra recorded for cellulose and for produced materials. (**a**) Cellulose (Cel); (**b**) Cel-DDTMABr; (**c**) Cel-TDTMABr; (**d**) Cel-HDTMACl; (**e**) Cel-DDTPPBr; (**f**) Cel-HDTBPBr; (**g**) Cel-MBT; (**h**) Cel-THIO.

In order to identify those functional groups specific to the extractants used for cellulose functionalization, cellulose and newly obtained materials FT-IR spectra were performed. These spectra are shown in Figure 3.

There are small differences due to the presence of active groups in the extractants. Table 1 shows the bands specific to the cellulose and extractant groups.

Based on the presented data, it can be stated that cellulose was successfully functionalized with the seven studied extractants. Small displacements of hydroxylic groups stretching vibrations observed in FT-IR spectra recorded for functionalized cellulose were associated with the formation of hydrogen bonds (physical intermolecular bonds) between used extractants and cellulosic free hydroxyl groups. This observation was in concordance with the low reactivity of cellulose in used working conditions, when only the cellulose swelling is taking place.

### 3.2. Studies on the Antimicrobial Activity of Materials

- Materials obtained by functionalizing cellulose with quaternary ammonium salts

A.1. Studies to determine the optimal cellulose:extractant ratio

The bacterial activity on chitosan can be considered intrinsic due to the presence of the amino group in its basic structure. The antibacterial activity of pure cellulose (avicel) can be highlighted only in its derivatives, obtained by various chemical functionalization with antibacterial groups or by combination with natural or synthetic bioactive components or polymers and with metal nanoparticles and metal oxides [21].

In order to establish the lowest cellulose:extractant ratio, which presents a bactericidal effect on the bacterial inoculum, each material was inoculated after incorporation into the culture medium and incubated, after which the total number of colonies was determined, according to the corresponding M1 non-functionalized cellulose (Cel) control sample, respectively the M0 control sample corresponding to the microbial inoculum. This ratio, for which the complete absence of microbial growth was observed, was considered the optimal, minimum required ratio, producing a total bactericidal effect. However, where microbial growth was observed onto the culture media, the significant antimicrobial effect was considered the highest calculated inhibition rate.

**Figure 4:**
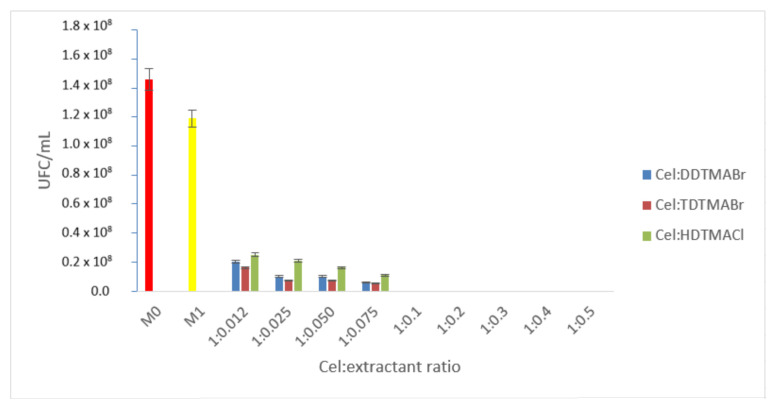
Comparison of the control sample (M0)—cellulose (M1)—cellulose:extractant (quaternary ammonium salts).

Analyzing the data presented in Figure 4, the non-functionalized cellulose sample (M1—yellow) has an insignificant antimicrobial effect compared to the control sample (M0—red). 

Regarding the three ammonium salts used as extractants, the best antimicrobial activity was highlighted by TDTMABr, in which case the ratio Cel:TDTMABr = 1:0.1 expresses the fact that the inhibition rate of bacterial growth was 100%. The DDTMABr extractant showed a total bactericidal effect when the ratio Cel:DDTMABr was 1:0.1 also. However, the inhibition rate of bacterial growth in the ratio of less than 1: 0.01 was lower for DDTMABr than for TDTMABr. For example, when cellulose:extractant ratio = 1:0.075 has been used, DDTMABr had an inhibition rate of 40%, while it was 95.8% for TDTMABr, respectively. HDTMACl extractant also showed total bactericidal effect at the functional ratio Cel:HDTMACl = 1:0.01. At the same time, the bacterial growth inhibition rate calculated for the HDTMACl extractant, at all functional ratios of less than 1:0.1, was lower compared to that calculated for TDTMABr and DDTMABr, respectively.

Consequently, the optimum functional ratio to express the total bactericidal effect in the case of quaternary ammonium salt extractants was considered when the cellulose:extractant ratio was 1:0.1.

A.2. Antimicrobial effect of ammonium materials on reference microbial strains

The most accessible groups present on the cellulose surface that contribute to the formation of new materials with antibacterial properties are the hydroxyl groups at the non-reducing end of the cellulose chain. Thus, the—OH groups on the crystalline cellulose were grafted with quaternary ammonium compounds through an adsorption process by SIR impregnation to allow a high concentration of quaternary ammonium groups on the cellulose surface. The effects of the obtained materials on four reference strains were tested. Table 2 shows the inhibition rate of microbial growth, calculated for each tested material.

Non-functional cellulose showed a slightly better bactericidal effect on Gram-negative bacteria compared to Gram-positive ones. All cellulose derivatives based on quaternary ammonium salts used in this study (DDTMABr, TDTMABr, HDTMACl) showed very good antibacterial activity on *S. aureus* and *C. albicans*, regardless of the functionalization ratio of the support material. This effect may be due to the similar behavior of the cell wall of *S. aureus* to the fungal cell of *C. albicans* [51,52]. The extractant strongly adheres to the bacterial cell wall and the fungal cell membrane, damaging their structure and preventing the exchange of nutrients necessary for microbial development, which subsequently leads to cell death, meaning the accomplishment of the antimicrobial effect. Another important role in the permeability of the cell membrane is the number of fatty acids present in its structure, which subsequently correlates with the degree of penetration of the toxic element into the cell, as shown in numerous studies [51,53].

Moreover, on the *E. coli* strain, the bactericidal effect was maximum when using DDTMABr and TDTMABr. At the same time, for HDTMACl, the highest inhibition rate was 67.5%, corresponding to the functionalization ratio Cel:HDTMACl = 1:0.1. A possible explanation would be that a particularly important role for antibacterial effect has the hydrophobic component given by the alkyl radical of the quaternary salt. On the other hand, there is the distance from the positive charge given by the quaternary nitrogen to the basic skeleton of the cellulose, respectively, the conformation of the alkyl substituent that forms the chain of the extractant and distances the hanging group. The length of the alkyl substituent, although differing only by two carbon atoms, in the order of dodecyl, tetradecyl, hexadecyl, tends to adopt different conformations [38], and implicitly the distance determined by the alkyl chain from the cellulose skeleton increases in the order of TDTMABr, DDTMABr, HDTMACl. Therefore, the strongest antibacterial effect has the TDTMABr extractant, regardless of the studied functionalization ratio. This statement is in line with the data in the literature, which claims that the structure of the cell wall is destroyed and cell viability is disrupted by the action of hydrophobic compounds [53,54,55,56,57]. 

Regarding the effect on *P. aeruginosa*, all tested ammonium salt extractants showed a bactericidal effect, correlated with the amount of extractant used for functionalization. The maximum inhibition rate was not reached at any of the studied ratios, and a much higher amount of biocide is likely to be required. The outer membrane of the bacterial cell in *P. aeruginosa* is an important selective barrier, which results in reduced adsorption of the biocide in the cell due to the reduced susceptibility of this species to biocidal agents [58].

Previous research results on quaternary ammonium cellulose salts have shown that they can be used successfully in wastewater treatment as flocculants [21,59,60]. According to this study, its use is also recommended in terms of the effectiveness of its antibacterial effect on wastewater.

B. Materials obtained by functionalization of cellulose with extractants containing phosphonium salts

B.1. Studies to determine the optimal cellulose:extractant ratio

**Figure 5:**
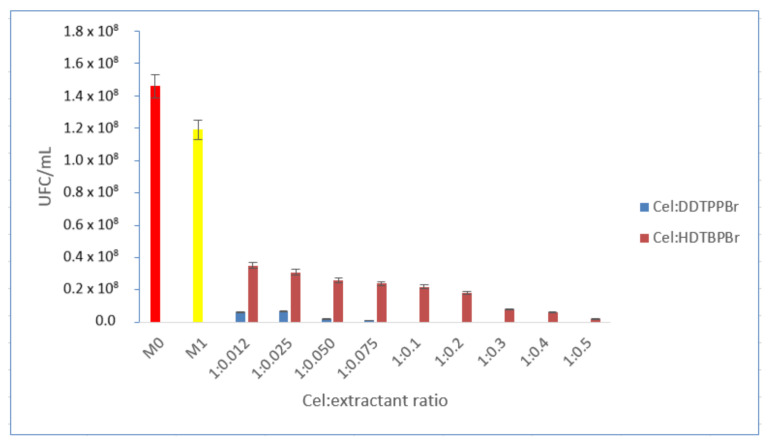
Comparison of the control sample (M0)—cellulose (M1)—cellulose:extractant (quaternary phosphonium salts).

Regardless of the phosphonium salt used for functionalization, the obtained material’s antimicrobial activity is directly dependent on the increase of the functionalization ratio (Figure 5).

When phosphonium salts were used as extractants, DDTPPBr showed a total antimicrobial effect at the functionalization ratio Cel:DDTPPBr = 1:0.1, while HDTBPBr showed a very good antimicrobial effect starting with the functional ratio Cel:HDTBPBr = 1:0.3, but did not show total bactericidal effect even at the ratio Cel:HDTBPBr = 1:0.5. Consequently, the optimal functionalization ratio for DDTPPBr is considered Cel:DDTPPBr = 1:0.1, and for HDTBPBr the optimal ratio is Cel:HDTBPBr = 1:0.5.

B.2. Antimicrobial effect of phosphonium materials on reference microbial strains

In the case of phosphonium salt extractants, they differ both in the length of the chain of the alkyl substituent grafted on the basic structure of the cellulose (dodecyl and hexadecyl) and in the different quaternization substituent (triphenyl and tributyl, respectively). The calculated microbial inhibition rates for each material studied are shown in Table 3. 

In the case of DDTPPBr, the antibacterial and antifungal effect was manifested with an inhibition rate of 100%, regardless of the functionalization ratio, except for the plates inoculated with *P. aeruginosa*. In the case of *P. aeruginosa*, the 10-fold increase in functional ratio did not lead to a substantial increase in the rate of bacterial inhibition, which increased only from 32% to 39.8%. This aspect can be explained by the resistance of *P. aeruginosa* species [58] to numerous agents with bactericidal effects and the need to use a quantity of extractant for functionalization that is not economically justified.

When using materials with HDTBPBr, the total bactericidal effect was obtained for *S. aureus* strain at the functionalization ratio Cel:HDTBPBr = 1:0.3. The total antifungal effect was obtained in the case of this material, also at the functionalization ratio Cel:HDTBPBr = 1:0.3. This could be explained by the resemblance of the cell walls of Gram-positive bacteria to the fungal wall, at least in terms of the wall structure of *C. albicans* species [51,52]. It was also found that the rate of inhibition of bacterial and fungal growth, respectively, is directly proportional to the functionalization ratio. In both Gram-negative bacteria tested, HDTBPBr failed to reach the maximum inhibition rate. On *P. aeruginosa*, even if the Cel:HDTBPBr functionalization ratio was increased 10-fold, from 0.01 to 0.1, the inhibition rate increased from 6% to 21.6%. Respectively, as the functional ratio increased from 0.1 to 0.5, the inhibition rate increased from 21.6% to 42.6%. The same effect was observed on the *E. coli* strain when the increase of the functionalization ratio by 50 times (from 0.01 to 0.5) determined only a doubling of the increase of the inhibition rate, from 42.7% to 89.1%. 

DDTPPBr is the extractant whose hydrophobicity is much higher than that of HDTBPBr because even if the alkyl substituent is shorter in the case of DDTPPBr (dodecyl versus hexadecyl), the phenyl substituent with which this extractant is quaternized has a high degree of hydrophobicity compared to the butyl substituent from HDTBPBr. On the other hand, being shorter, the alkyl segment causes a smaller distance of the pendant group from the basic structure of cellulose, which again increases the antimicrobial effect. All these findings are supported by data from the literature that emphasize the role of the hydrophilic-hydrophobic balance of the bacterial cell wall and the hydrophobicity of the antibacterial agent in the manifestation of the bactericidal effect [53,55,56,61]. Moreover, the electrostatic attraction between the functionalized cellulose and the bacterial cell membrane plays an essential role in the bactericidal effect, as the strong electrostatic attraction forces in the adsorbed polyelectrolyte and the lipid molecules in the bacterial cell membrane ultimately destroy the bacterial cell [61,62].

C. Materials obtained by functionalizing cellulose with sulfur compounds

C.1. Studies to determine the optimal cellulose:extractant ratio

**Figure 6:**
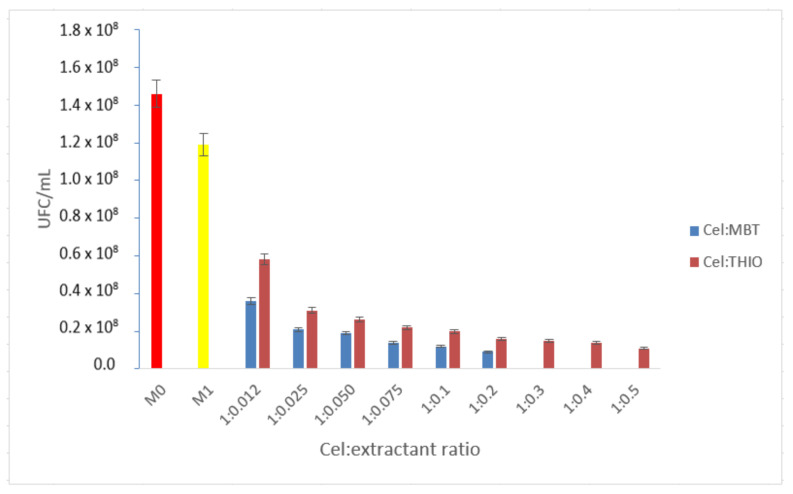
Comparison of the control sample (M0)—cellulose (M1)—cellulose:extractant (compounds containing sulfur).

Compared to the analysis of the M1 control sample, when non-functionalized cellulose allowed the growth of bacteria on the surface of the culture medium, it can be concluded that by functionalizing cellulose with MBT and THIO, increasing functionalization ratio, bactericidal capacity is positively influenced (Figure 6).

After examining the series of microbial cultures in which MBT was used as an extractant, it was found that there was no microbial growth at the ratio Cel:MBT = 1:0.3; there was no microbial growth. Thus, the ratio that showed total bactericidal effect and is considered as optimal working ratio is 1:0.3.

For the THIO extractant, regardless of the studied functionalization ratio, no total bactericidal effect was obtained, proven by the maximum calculated bactericidal inhibition rate of 90.8% for the functionalization ratio of 1:0.5. In order to establish the optimal working ratio, given that we cannot talk about the total bactericidal effect, we considered the optimal ratio Cel:THIO = 1:0.5.

C.2. Antimicrobial effect of sulfur materials on reference microbial strains

The efficiency of the materials obtained by functionalizing cellulose with sulfur-containing extractants is expressed, in the form of the rate of microbial inhibition, in Table 4.

The functionalization of cellulose with MBT or THIO has led to the improved antibacterial and antifungal activity of native cellulose, proven by obtaining a higher inhibition rate for Cel-MBT and Cel-THIO materials than native cellulose, even for the minimum functionalization ratio Cel:extractant = 1:0.012. 

We compared the efficiency of the functionalized material with MBT on the tested species. We find that the antimicrobial effect is better in the case of Gram-positive bacterial and fungal species, proven by the 100% inhibition rate achieved for a functionalization ratio Cel:MBT = 1:0.1 for both *S. aureus* and *C. albicans*. The similar manifestation of the antimicrobial effect exerted by the two species under the action of the MBT-based materials tested is suggestive, as the inhibition rate calculated for the functional ratio 1:0.05 is approximately equal (62.9% for *S. aureus* and 63.2% for *C. albicans*). When the effect of these sulfur-containing materials on Gram-negative bacteria was studied, their efficacy was found to be directly related to the increase in the amount of extractant and to the increase in functionalization ratio, but the maximum bacterial inhibition rate was obtained only for *E. coli* at a ratio Cel:MBT = 1:0.5. The functionalization of cellulose with THIO, even at the highest functionalization ratio, had no total antimicrobial effect. We could rather speak of the bacteriostatic or fungistatic effect in the case of materials functionalized with thiourea. Suppose we consider sufficient the manifestation of the bacteriostatic or fungistatic effect. In that case, it is found that the newly synthesized biomaterial prevented the growth of bacteria without necessarily killing them, observing an inactivation of microbial strain growth, as shown by the analysis of inhibition rates presented in Table 4. 

In Table 5, we synthesized a comparison between the antimicrobial effect (regarding the inhibition of microbial growth) of the materials synthesized in the present paper and other studies.

## 4. Conclusions

In the context of the resistance of microbial strains to most bactericidal agents known to date, the development of new antibacterial materials or at least with bacteriostatic properties, starting from biomaterials, has opened new opportunities to inhibit microbial adhesion and limit their transmission. As the polyolic nature of cellulose allows it to bind a series of compounds with chemical functions that can modulate the hydrophilic/lipophilic balance so that it can provide improved properties, we are determined to consider this biopolymer as a good support material applicable in many fields. For example, with regard to the use of cellulose derivatives in food preservation technology, the identification of new biomaterials with antibacterial properties is one of the most promising trends. It is even desirable to obtain such antibacterial materials that are biodegradable, which is why the study of cellulose is desirable. Moreover, the antimicrobial potential of cellulose functionalized in wastewater treatment should not be neglected, especially in terms of water disinfection, as evidenced by the results obtained in this study.

The impregnation technique used in this paper for the functionalization of cellulose is an easy way to obtain cellulose derivatives without involving the production of reaction by-products. On the other hand, in the case of all cellulose derivatives obtained by impregnation, increased antimicrobial properties were obtained compared to native cellulose, which leads to the conclusion that this impregnation technique can be used successfully to obtain new materials with very good antimicrobial effect.

Growth inhibition of *S. aureus*, *E. coli,* and *C. albicans* strains was determined by using materials with DDTMABr, TDTMABr, and DDTPPBr, regardless of the functionalization ratio. Materials obtained by functionalizing cellulose with quaternary ammonium and phosphonium salts showed substantial antibacterial capacity against *S. aureus*, *E. coli*, *C. albicans* (up to 100%), and *P. aeruginosa* (maximum 54.8%) even at very low concentrations of surface-immobilized antimicrobial agent. In the case of materials obtained by functionalization with quaternary ammonium salts, the antibacterial activity was dependent on the alkyl chain’s length, which distances the quaternary salt’s pendant group from the basic structure of the cellulose. In this sense, the antimicrobial effect was proved in the order of TDTMABr > DDTMABr > HDTMACl, respecting at the same time the increase of the ratio between the support material and extractant.

In the case of materials obtained by the functionalization of cellulose with phosphonium quaternary salts, the antibacterial activity was strongly influenced by the hydrophobic character of the pendant group (phenyl compared to butyl), in addition to the distance given by the alkyl substituent of the quaternary salt (dodecyl compared to hexadecyl) was added. The best antimicrobial effect was observed when it was used DDTPPBr like extractant, even at lower support:extractant ratio = 1:0.012.

Materials obtained by functionalizing cellulose with sulfur extractants have generally shown a bacteriostatic or fungistatic effect, with a maximum of 76% inhibition rate upon *S. aureus* at the highest functionalized support:extractant ratio = 1:0.5. The exception was in the case of Cel-MBT when the maximum inhibition rate on *S. aureus* and *C. albicans* was reached at the functionalization ratio Cel:MBT = 1:0.3.

The abundance of polysaccharides can effectively prepare antibacterial materials either by enhancing or modulating natural antibacterial properties (such as chitosan) or by de novo imparting these properties to cellulose or other biopolymers makes these biomaterials increasingly often studied, and their applicability increasingly varied. The combination of antibacterial activity with other well-known properties of these polysaccharides opens up good perspectives for the future development of such innovative functional materials.

## Figures and Tables

**Table 1:** FT-IR specific bands in cellulose and extractants [49,50].

Group	FT-IR Bands (cm^−1^)	Observations
Cellulose (Cel)		
O-H C-H CH_2_ C-O O-C-O OH_2_	3660 2893 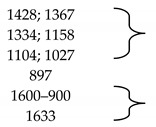	Large band Small plateau; stretching vibrations in polysaccharides Vibrations specific to the crystalline structure of cellulose Amorphous region in cellulose Water molecules vibrations
Cel-DDTMABr		
>N-CH_2_	2700–2800	specific link e^−^ nonparticipants from N
Cel-TDTMABr		
>N-CH_2_	2700–2800	specific link e^−^ nonparticipants from N
Cel-HDTMACl		
>N-CH_2_	2700–2800	specific link e^−^ nonparticipants from N
Cel-DDTPPBr		
P-O-Aril C-O (fenil) O-H	1190–1240 1200 3500–3200	
Cel-HDTBPBr		
P-O-Alchil	1150–1180; 1080	
Cel-MBT		
S-C-S C-N C-H; N-H	568–600 1030–1074 1250–1320 750	Aromatic ring -torsion Stretching vibration
Cel-THIO		
-NH_2_ N-H C=S	3395 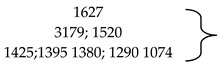	Asymmetric vibration; symmetric vibration Elongation vibration Vibrations strongly influenced by intramolecular effects

**Table 2:** Antimicrobial growth inhibition rate when using ammonium salts materials.

Material	Cel:Extractant Ratio	Inhibition Rate (%)				OBSERVATIONS
		*Staphylococcus aureus ATCC 25923*	*Pseudomonas aeruginosa ATCC 27853*	*Escherichia coli ATCC 25922*	*Candida albicans ATCC 10231*	
Cellulose (Cel)	-	2.8	7.7	20	12.2	Slightly better bactericidal effect on Gram-negative bacteria and *C. albicans* strain
Cel:DDTMABr	1:0.012	100.0	40.8	100.0	100.0	Maximum bactericidal effect on Gram-positive bacteria and *C. albicans* strain
	1:0.1	100.0	52.8	100.0	100.0	
Cel:TDTMABr	1:0.012	100.0	49.9	100.0	100.0	
	1:0.1	100.0	54.8	100.0	100.0	
Cel:HDTMACl	1:0.012	100.0	17.0	53.4	100.0	
	1:0.05	100.0	34.3	58.1	100.0	
	1:0.1	100.0	40.8	67.5	100.0	

**Table 3:** Inhibition rate of microbial growth when using phosphonium materials.

Material	Cel:Extractant Ratio	Inhibition Rate (%)			
		*Staphylococcus aureus ATCC 25923*	*Pseudomonas aeruginosa ATCC 27853*	*Escherichia coli ATCC 25922*	*Candida albicans ATCC 10231*
Cel:DDTPPBr	1:0.012	100.0	32.0	100.0	100.0
	1:0.1	100.0	39.8	100.0	100.0
Cel:HDTPPBr	1:0.012	30.7	8.4	42.7	65.3
	1:0.05	58.7	20.7	44.9	67.0
	1:0.1	69.7	21.6	52.6	77.9
	1:0.3	100.0	26.6	87.3	100.0
	1:0.5	100.0	42.6	89.1	100.0

**Table 4:** Microbial growth inhibition rate when using sulfur materials.

Material	Cel:Extractant Ratio	Inhibition Rate (%)			
		*Staphylococcus aureus ATCC 25923*	*Pseudomonas aeruginosa ATCC 27853*	*Escherichia coli ATCC 25922*	*Candida albicans ATCC 10231*
Cel:MBT	1:0.012	25.6	10.0	20.5	54.0
	1:0.05	62.9	13.7	22.1	63.2
	1:0.1	100.0	19.5	50.4	100.0
	1:0.3	100.0	36.2	65.4	100.0
	1:0.5	100.0	41.0	100.0	100.0
Cel:THIO	1:0.012	20.7	12.9	28.9	52.5
	1:0.05	27.7	20.7	49.6	55.8
	1:0.1	39.3	39.6	51.0	54.5
	1:0.3	75.3	44.5	52.4	60.1
	1:0.5	76.0	45.1	55.5	60.9

**Table 5:** Comparison between cellulose derivative and antimicrobial effect.

Material	Antimicrobial Effect (Inhibition Rate %) upon Microbial Strain	References
Cellulose with octadecyldimethyl(3-trimethoxysilylpropyl) ammonium chloride	96–99% on *E. coli* >99% on *S. aureus*	[63]
Silver–cellulose fiber sheets	91.58–99.46% on *S. aureus* 93.6–99.78% on *E. coli* 97.5–100% on *C. albicans*	[43]
Propane sulfonated chitosan	78.8% on *Phomopsis asparagi* 80.2% on *Fusarium oxysporum*	[44]
Dipropane sulfonated chitosan	82.2% on *Phomopsis asparagi* 94% on *Fusarium oxysporum*	[44]
Gold nanoparticles	5.4–20.0% on *E. coli* 4.6–16.3% on *S. aureus*	[45]
Gold nanocapsules	23.7–40.0% on *E. coli* 18.6–34.9% on *S. aureus*	[45]
Chitosan derivatives based upon quaternary ammonium salt	100% on *S. aureus* * 13.1–60.3% on *P. aeruginosa* * 25.8–100.0% on *E. coli* * 76.0–100.0% on *C. parapsilosis* *	[38]
Chitosan derivatives based upon phosphonium salt	100% on *S. aureus* * 28.2–48.6% on *P. aeruginosa* * 20.2–100.0 on *E. coli* * 100.0% on *C. parapsilosis* *	[38]
Chitosan derivatives based upon sulfur compound	38.6–100% on *S. aureus* * 22.8–46.7% on *P. aeruginosa* * 25.8–72.3% on *E. coli* * 80.6–100.0% on *C. parapsilosis* *	[38]
Cellulose derivatives based upon quaternary ammonium salt	100.0% on *S. aureus* * 17.0–54.8% on *P. aeruginosa* * 53.4–100.0% on *E. coli* * 100% on *C. albicans* *	present study
Cellulose derivatives based upon phosphonium salt	30.7–100.0% on *S. aureus* * 8.4–42.6% on *P. aeruginosa* * 42.7–100.0% on *E. coli* * 65.3–100.0% on *C. albicans* *	present study
Cellulose derivatives based upon sulfur compound	20.7–100.0% on *S. aureus* * 10.0–45.1% on *P. aeruginosa* * 20.5–100.0% on *E. coli* * 52.5–100.0% on *C. albicans* *	present study

* inhibition rate value is depending on the functionalization ratio and the tested material.

## Data Availability

Not applicable.
